# The bodily self from psychosis to psychedelics

**DOI:** 10.1038/s41598-023-47600-z

**Published:** 2023-12-01

**Authors:** Amir Harduf, Gabriella Panishev, Eiran V. Harel, Yonatan Stern, Roy Salomon

**Affiliations:** 1 The Multidisciplinary Brain Research Center, Bar-Ilan University, 5290002 Ramat-Gan, Israel; 2 The Faculty of Life Sciences, Bar-Ilan University, 5290002 Ramat-Gan, Israel; 3 Beer Yaakov-Ness Ziona Mental Health Center, Beer Yaakov, Israel; 4 Department of Cognitive Sciences, University of Haifa, 3498838 Haifa, Israel

**Keywords:** Psychology, Human behaviour, Diseases, Psychiatric disorders

## Abstract

The sense of self is a foundational element of neurotypical human consciousness. We normally experience the world as embodied agents, with the unified sensation of our selfhood being nested in our body. Critically, the sense of self can be altered in psychiatric conditions such as psychosis and altered states of consciousness induced by psychedelic compounds. The similarity of phenomenological effects across psychosis and psychedelic experiences has given rise to the “psychotomimetic” theory suggesting that psychedelics simulate psychosis-like states. Moreover, psychedelic-induced changes in the sense of self have been related to reported improvements in mental health. Here we investigated the bodily self in psychedelic, psychiatric, and control populations. Using the Moving Rubber Hand Illusion, we tested (N = 75) patients with psychosis, participants with a history of substantial psychedelic experiences, and control participants to see how psychedelic and psychiatric experience impacts the bodily self. Results revealed that psychosis patients had reduced Body Ownership and Sense of Agency during volitional action. The psychedelic group reported subjective long-lasting changes to the sense of self, but no differences between control and psychedelic participants were found. Our results suggest that while psychedelics induce both acute and enduring subjective changes in the sense of self, these are not manifested at the level of the bodily self. Furthermore, our data show that bodily self-processing, related to volitional action, is disrupted in psychosis patients. We discuss these findings in relation to anomalous self-processing across psychedelic and psychotic experiences.

## Introduction

The self is a central organizing principle of our cognition and experience. The sense of self is composed of different levels or models^1–6^ subserved by different neural systems^7–13^, which in neurotypical states are manifested as a robust and unified phenomenological experience. Altered states of consciousness, such as those found in neurological, psychiatric, and psychedelic states, are known to alter this experience e.g.,^14–16^. Notably, psychosis and psychedelic states both produce hallucinations and substantial alterations in the sense of self e.g.,^17–23^, causing some researchers to consider the psychedelic experiences as psychotomimetic^24,25^. For example, psychedelic experiences may induce changes across many levels of self, from the narrative self^26,27^ to robust changes in the ‘minimal self’^28^. Changes in minimal self may include abnormal feelings of ownership over one’s body, aberrant bodily sensations, loss of spatial self-location, and even a loss of the sense of self—“ego dissolution”^15,18,29,30^. Indeed, “ego dissolution” is an extreme condition associated with high doses of psychedelics leading to a loss of segregation between oneself and one's surroundings and typically accompanied by a feeling of unity with the universe^15,31^.

Similarly, the disrupted self is a prominent feature in psychosis^32–38^. Schizophrenia patients show deficits across different levels of self, including narrative^39,40^, social^41^, and bodily^23,34,42,43^ models of the self. Positive symptoms in schizophrenia include diminished control over one’s thoughts and actions, which lead to “passivity” experiences like auditory hallucinations and thought insertion^23,43,44^. It has been proposed that abnormal predictive sensorimotor processes blur the demarcation of self-generated sensations and drive self-deficits in psychosis^34,45,46^. This, in turn, may cause one’s inner voice, thoughts, and actions to be felt as if they are generated by some external agent leading to symptoms found in psychosis^22,43,47,48^. Thus, both psychedelic and psychotic states are unique examples in which the fundamental dimensions of the bodily self are altered.

The bodily self consists of two fundamental aspects, Body Ownership, the experience of identifying with the body e.g.,^49–51^, and the Sense of Agency, the experience of control over one’s actions e.g.,^5,43,52–57^. Body Ownership relies on multisensory integration in which exteroceptive and interoceptive sensory signals are bound to form a coherent model of the self e.g.,^5,12,58–60^. For example, when the brain receives converging visual, tactile, and proprioceptive signals congruent with prior implicit models of the self, a feeling of ownership arises for this limb. The Sense of Agency is grounded in predictive motor and inference processes linking volitional actions to the predicted sensory outcomes e.g.,^57,61,62^. Previous work has shown that voluntary actions are accompanied by an efference copy, which is used to compute forward models predicting the expected sensory consequences e.g.,^63–65^. The comparison of the predicted and actual sensory feedback enables the agent to distinguish between internally and externally generated sensations. If they match, a feeling of a self-generated movement arises (that the action is self-generated), and this is accompanied by both behavioral^66–68^ and neural^69–72^ suppression of the sensory consequences. However, there are documented instances where actions have led to sharpened sensory information^73–75^. If there is a large discrepancy between an action's predicted and actual sensory outcomes, the action is not attributed to oneself. For example, if the visual outcome of an action is modified temporally e.g.,^76–78^, anatomically e.g.,^56^, or spatially e.g., ^79–81^, participants do not experience authorship of the action. Thus, both Body Ownership and Sense of Agency rely on integration of sensory signals, however, Sense of Agency utilizes information from volitional action which can be used to improve inferences on the bodily self^82^.

The experimental study of the bodily self has relied heavily on the induction of illusory states of Body Ownership and Sense of Agency. In their seminal work, Botvinick and Cohen revealed that Visuotactile stimulation on a visible rubber hand, synchronous to tactile stimulation on one’s unseen real hand, induces illusory ownership over the rubber hand^83^. The rubber hand illusion, as well as full body and virtual versions of this paradigm, have become a mainstay of experimental research on the bodily self e.g.,^1,13,84–86^. Indeed, numerous subsequent studies have shown that induction of illusory Body Ownership is accompanied by replicable behavioral, physiological, and neural states^87–90^. Sense of Agency is typically tested by introducing discrepancies between participants’ movements and their visual consequences. For example, several studies have used temporal or spatial visuomotor discrepancies to alter and decrease the experience of Sense of Agency e.g.,^56,76,86,91^. Most experiments have focused on either Body Ownership or Sense of Agency such that their interactions were neglected. The Moving Rubber Hand illusion, a paradigm introduced a decade ago by Kalckert and Ehrsson allowed examining both Body Ownership and Sense of Agency by introducing both Visuotactile and Visuomotor illusions in a within-subject design^92,93^.

Here we used the Moving Rubber Hand illusion to examine alterations in the bodily self across participants with substantial psychedelic experience, participants with psychosis, and neurotypical controls. In psychosis, there is substantial evidence of impairments in Sense of Agency^22,34,94^. Across different paradigms, participants on the psychosis spectrum show a reduced ability to discriminate self-initiated actions^95^. Recent evidence has shown that this deficit is even present in non-symptomatic 22Q11DS participants with a genetic propensity for psychosis, suggesting Sense of Agency deficits may be a precursor of the psychotic state^38^. This aberrant Sense of Agency has been suggested to stem from abnormal predictive sensorimotor processes, which have been causally shown to induce psychosis-like states^14,96^. While there is converging evidence for Sense of Agency deficits in psychosis, the state of Body Ownership is less clear. While early reports reported abnormal processing of the Rubber Hand illusion in schizophrenia, recent empirical and meta-analytical work has shown no differences between control and schizophrenia participants for Body Ownership^97^. Classic psychedelics, which have been suggested to mimic psychosis symptoms^98,99^, also induce striking alterations of self, including strange sensations of the bodily self e.g.,^30,100^. These have been suggested to drive by changes in neural connectivity primarily due to the activation of 5HT2A receptors^24,101–103^. However, while subjective reports of acute and long-term changes in the sense of self after psychedelic use exist^104,105^, experimental evidence is conspicuously lacking. In this experiment, we compared Body Ownership and Sense of Agency using the Moving Rubber Hand illusion in participants with psychosis, participants with a history of substantial psychedelic experiences, and neurotypical controls.

## Materials and methods

### Participants

Seventy-five, right-handed participants took part in the experiment (27 females; Age M = 29.9, SD = 8.85). There were three groups of participants: *Control* (N = 25; 16 females; Age M = 24.84, SD = 6.28), *Psychedelic* (N = 25; 11 females; Age M = 28.8, SD = 6.01), and *Psychosis* (N = 25; all males; Age M = 36.08, SD = 10.39). The sample size was determined by previous studies using the Rubber Hand Illusion or the Moving Rubber Hand Illusion with similar sample sizes^106–110^. The *Control* and *Psychedelic* groups included participants who self-reported no history of neurological, psychiatric, or tactile and motor disorders and any use of associated medications. Participants in the *Psychedelic* group were collected by approaching psychedelic social media groups and all participants reported psychedelic substance use, whereas in the Control group, they reported no use (see Supplementary Fig. S2). In the psychedelic cohort 50% reported having experienced psychedelics over 15 times and about 25% reporting having used psychedelics over 50 times. The *Psychosis* group included patients with psychosis hospitalized at the Beer Yaakov-Ness Ziona Mental Health Center (see Supplementary Table S4). The experiment was performed following the ethical standards of the Declaration of Helsinki and was approved by the Gonda Multidisciplinary Brain Research Center ethics committee (for the *Control* and *Psychedelic* groups) and by the Beer Yaakov-Ness Ziona Mental Health Center ethics committee (for the *Psychosis* group). All participants gave written informed consent before the experiment. The *Psychosis* group had a higher mean age than other groups, Wilcoxon rank-sum test (*Psychosis* vs. *Control*: W = 505.5, *p* < 0.001; *Psychosis* vs. *Psychedelic*: W = 447.5, *p* < 0.01).

### Assessment of psychedelic use and changes in Self

The *Psychedelic* and *Control* groups self-reported their frequency of use of psychedelic substances as well as other substances (e.g., alcohol and other compounds see Supplementary Fig. S2–S6). We wished to assess changes in the Sense of Self and especially the bodily self, due to psychedelic experiences. We thus chose to rely on previously established questionnaires, while focusing on items particularly relevant to the study’s main aim. Specifically, to assess changes in the sense of self following psychedelic use, we constructed a questionnaire examining altered self-experiences using 18 statements gathered from several well-established questionnaires (Ego-Dissolution Inventory (EDI)^31^, Cambridge Depersonalization Scale (CDS)^111^, and the Pahnke-Richards Mystical Experience Questionnaire (MEQ)^112^ that examine the frequency and duration of experiences such as depersonalization, ego-dissolution, internal and external unity, and transcendence of time and space. These included items such as “Whilst doing something I have the feeling of being a detached observer of myself” and “I cannot feel properly the objects that I touch with my hands for it feels as if it were not me who was touching it” measuring depersonalization. Other items such as “I experienced a disintegration of my self or ego” and “All notion of self and identity dissolved away” probed other aspects such as ego-dissolution (see Supplementary Table S2 for full questionnaire details). The questionnaire was completed by both the *Psychedelic* and *Control* groups. To assess whether psychedelic use is associated with increased altered self-experiences, we compared the questionnaire scores for each section (EDI, CDS, and MEQ) between the *Psychedelic* and *Control* groups using a Mann–Whitney test. *Psychosis* patients’ clinical symptoms were assessed by a clinician via the Positive and Negative Syndrome Scale (PANSS) (see the clinical characteristics of *Psychosis* patients in Supplemental Table S4).

### Experimental setup

**Figure 1:**
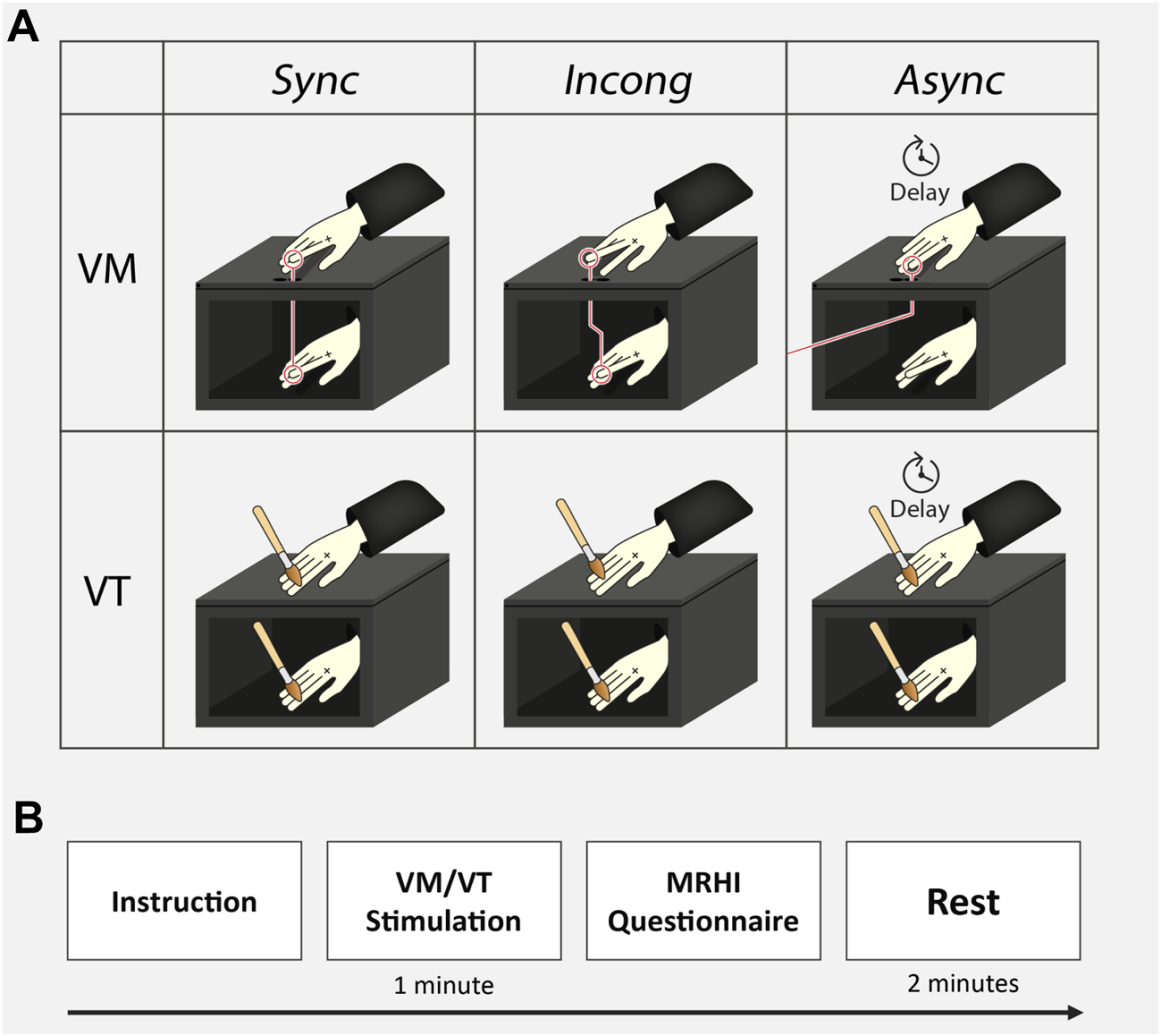
Experimental paradigm. (**A**) Experimental design illustration of Visuomotor (VM) and Visuotactile (VT) stimulations, each divided for the *Sync* (synchronous coupling/stroking on both index fingers), *Incong* (synchronous coupling/stroking on the subject’s index finger and the rubber hand’s middle finger), and *Async* (coupling/stroking on both index fingers with a temporal delay) conditions. Red connecting lines in Visuomotor represent the mechanical connection between the subject’s index finger and the rubber hands index finger in *Sync*, the middle finger in *Incong*, or the experimenter’s finger for a delayed movement (*Async*). (**B**) Experimental procedure of a single run. In each run a single stimulation type (Visuotactile / Visuomotor) and condition (*Sync* / *Incong* / *Async*) was administered, after which the subject completed the Moving Rubber Hand illusion (MRHI) questionnaire that consists of 12 statements probing Sense of Agency and Body Ownership. The order of runs was randomized between subjects.

We used an adaptation of the Moving Rubber Hand illusion setup to assess both Body Ownership and Sense of Agency^92,93^. Participants sat at a table with their right hand hidden from their view and placed inside a box (35 cm × 25 cm × 12 cm). A realistic life-size and gender-matched rubber hand model was placed on top of the box and covered with a latex glove identical to the participants’ glove. The participant’s forearm was covered with a soft black cloth to ensure visual continuity of the rubber hand with the participant's arm (Fig. 1A).

### Experimental design

Participants underwent two types of sensorimotor stimulation: Visuotactile and Visuomotor. In the Visuotactile stimulation, as used in classical Rubber Hand illusion experiments to induce illusory Body Ownership^83^, participants were instructed to relax their hand while the experimenter brushed both the real and rubber hand with small brushes. In the Visuomotor stimulation, used in Moving Rubber Hand illusion experiments to elicit an illusion of Body Ownership and Sense of Agency over the rubber hand^89,92,93^, participants raised their index finger at a semi-regular rhythm of 1 Hz, which was practiced before the experiment. This movement resulted in a movement of the rubber hand by thin metal rod hidden inside the box. In both Visuotactile and Visuomotor stimulations, three different conditions were used. The brush strokes (i.e., Visuotactile) or active movement (i.e., Visuomotor) of the participant’s index finger was accompanied by either anatomically congruent and temporally synchronous stimulation of the rubber hand’s index finger (*Sync*), or anatomically incongruent and temporally synchronous stimulation of the rubber hand’s middle finger (*Incong*), or anatomically congruent and asynchronous stimulation with a 500 ms delay inserted between stimulation of the real and rubber hand (*Async*) (Fig. 1). In the Visuomotor *Async* condition, the metal rod was connected to the experimenter’s finger, allowing the experimenter to manipulate the rubber hand’s movement in a matter unknown to the participant. Each combination of stimulation type and condition was induced in a separate run lasting one minute, resulting in a total of six runs presented in a pseudorandomized order.

After each run, participants completed the Moving Rubber Hand illusion questionnaire^93^ which assesses their subjective experiences of Body Ownership and Sense of Agency. The questionnaire comprises 12 statements, including three statements referring to Body Ownership, three to Sense of Agency, three Body Ownership control statements, and three Sense of Agency control statements (see Supplementary Table S1 for the full list of statements). The control statements in the questionnaire do not capture the specific phenomenological experiences of Body Ownership or Sense of Agency, but rather have several similarities to the illusion-specific statements in general^92^. Each statement was rated on a 7-point Likert scale ranging from −3 “totally disagree” to + 3 “totally agree”, with 0 indicating “neutral”. *Control* and *Psychedelic* participants were presented with the statements on a computer screen and responded with a num-pad, while the *Psychosis* participants responded verbally.

### Data analysis

Statistical analyses were performed using JASP 0.16.3^113^ and R^114^. The Shapiro–Wilk test was used to assess normality (*p* > 0.05). Since the data sets failed to meet the criteria for normal distribution, the appropriate non-parametric tests were applied. Friedman’s repeated measures ANOVA was used to estimate the main effects of Condition (*Sync / Incong / Async*) as a within-subjects factor, with a Pairwise Wilcoxon Rank sum test (Bonferroni corrected) as post-hoc. One-way Kruskal–Wallis ANOVA was used to estimate the main effects of Group (*Control / Psychedelic / Psychosis*) as a between-subjects factor, with Dunn test (Bonferroni corrected) as post-hoc. To examine whether the effect of Group is modulated by Condition type, we calculated the differences between all Condition scores and conducted a One-way Kruskal–Wallis ANOVA on these differentials^115–117^. For non-significant results of the Group factor, we performed a Bayesian Mann–Whitney test between *Control* and *Psychedelic,* and between *Control* and *Psychosis,* to assess the evidence’s strength of the null hypothesis. Specifically, we used a Bayes factor of exclusion. Briefly, BF exclusion was obtained using JASP, by comparing the evidence of models that included the factor of interest to the evidence of models that did not include the factor. Since there was no prior knowledge on the effect between groups, we used the default prior (Cauchy = 0.707) and reported the robustness of the findings (See supplementary Table S3) using a wide prior (Cauchy = 1.414) and a narrow prior (Cauchy = 0.353)^118^. The models that were compared were of similar complexity, including the same number of variables^119^. Thus, BF exclusion represents how likely it is to observe the data under models that exclude the factor compared to models that include this factor. BF values between 1 and 3, 3 and 10, and 10 and 30, were interpreted as anecdotal, moderate, and strong evidence for the hypothesis respectfully^120^. To examine if a condition induced an illusion of Body Ownership or Sense of Agency over the rubber hand, we conducted a Wilcoxon signed-rank test by comparing the participants’ ratings to 0 (i.e., neutral experience). Conversely, the control ratings were compared to 0 in the opposite direction using a Wilcoxon signed-rank test, to ensure all were rated negatively below zero. To estimate the difference in Body Ownership illusion strength between the *Healthy* group (*Control* and *Psychedelic)* and the *Psychosis* group*,* we performed a Mann–Whitney test. The illusion strength for each group was computed as the difference in Body Ownership subjective rating between *Sync* and *Async* conditions. The *Sync* and *Async* conditions were also compared using a paired Wilcoxon signed-rank test within groups. We used *Spearman’s* correlations to estimate the association between the strength of Sense of Agency and Body Ownership illusion in each simulation. Comparing illusion scores between recent users (within the last month) and past users (beyond the last month) revealed no significant differences in scores for any of the illusion conditions; hence, this aspect was not further analyzed.

## Results

### Psychedelic experience is associated with altered self-experiences

**Figure 2:**
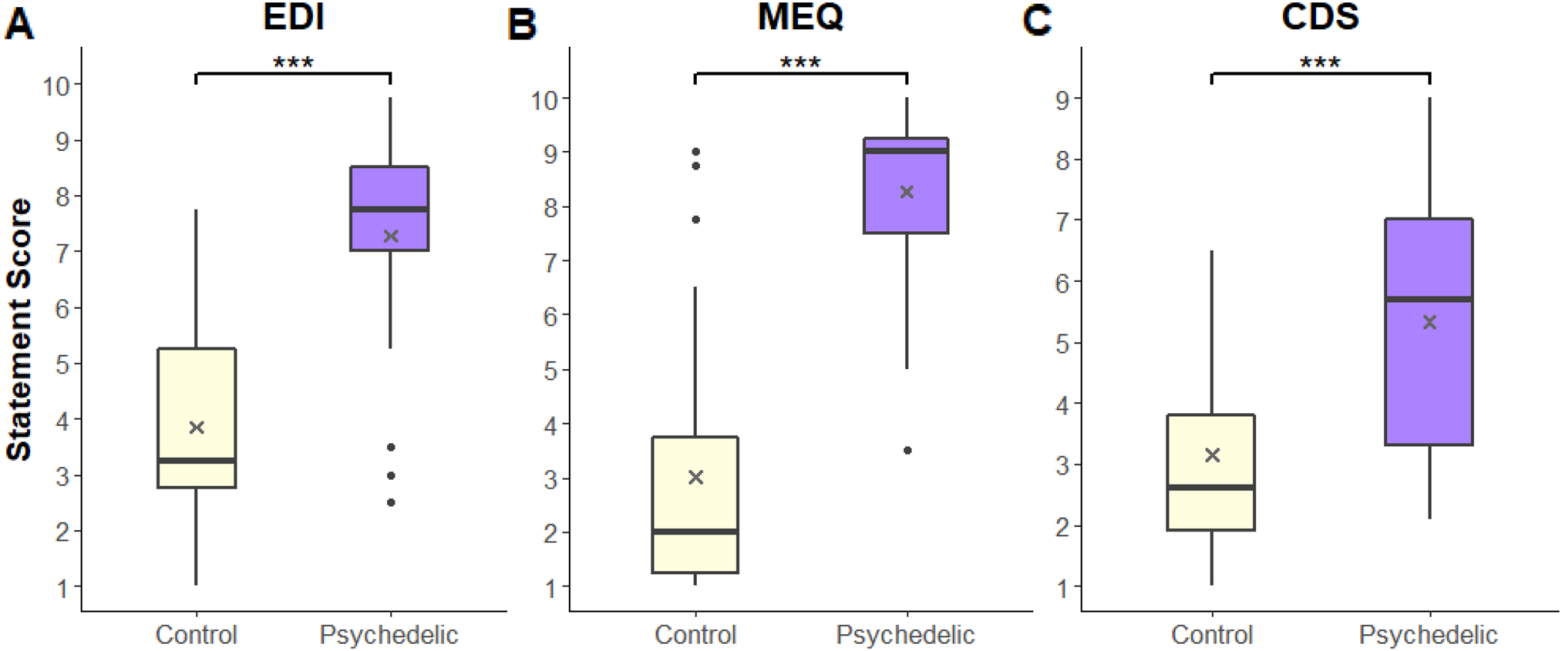
Psychedelic experience is associated with reported altered self-experiences. Statements scores were gathered from each questionnaire. (**A**) Scores for statements from Ego Dissolution Inventory (EDI). (**B**) Pahnke-Richards Mystical Experience Questionnaire (MEQ). (**C**) Cambridge Depersonalization Scale (CDS). Gray ‘X’ at the boxplots represents the group’s mean. Asterisks indicate significance levels: ∗ *p* < 0.05; ∗  ∗ *p* < 0.01; and ∗  ∗  ∗ *p* < 0.001.

To examine reported differences in self-related experiences between the *Control* and *Psychedelic* groups, we compared the mean scores of their statements across sections of the self-experience questionnaire Fig. 2. The *Psychedelic* group’s mean in the EDI statements (M = 7.28, SD = 2) was significantly higher than the *Control* group’s mean (M = 3.85, SD = 1.92) (W = 68, *p* < 0.0001). Similarly, in the MEQ statements, the *Psychedelic* group showed a higher mean (M = 8.27, SD = 1.65) than the *Control* group (M = 3.02, SD = 2.45) (W = 38, *p* < 0.0001; For the analysis of each question, see Supplementary Fig. S1 and Table S2). Additionally, in the CDS statements the *Psychedelic* group’s mean score (M = 5.33, SD = 1.99) was higher than the *Control* (M = 3.16, SD = 1.77) (W = 127, *p* < 0.001). These results support previous reports of the association between psychedelic experiences and changes in the sense of self^26,27,29,121^.

### Reduced sense of agency during visuomotor stimulation in psychosis

**Figure 3:**
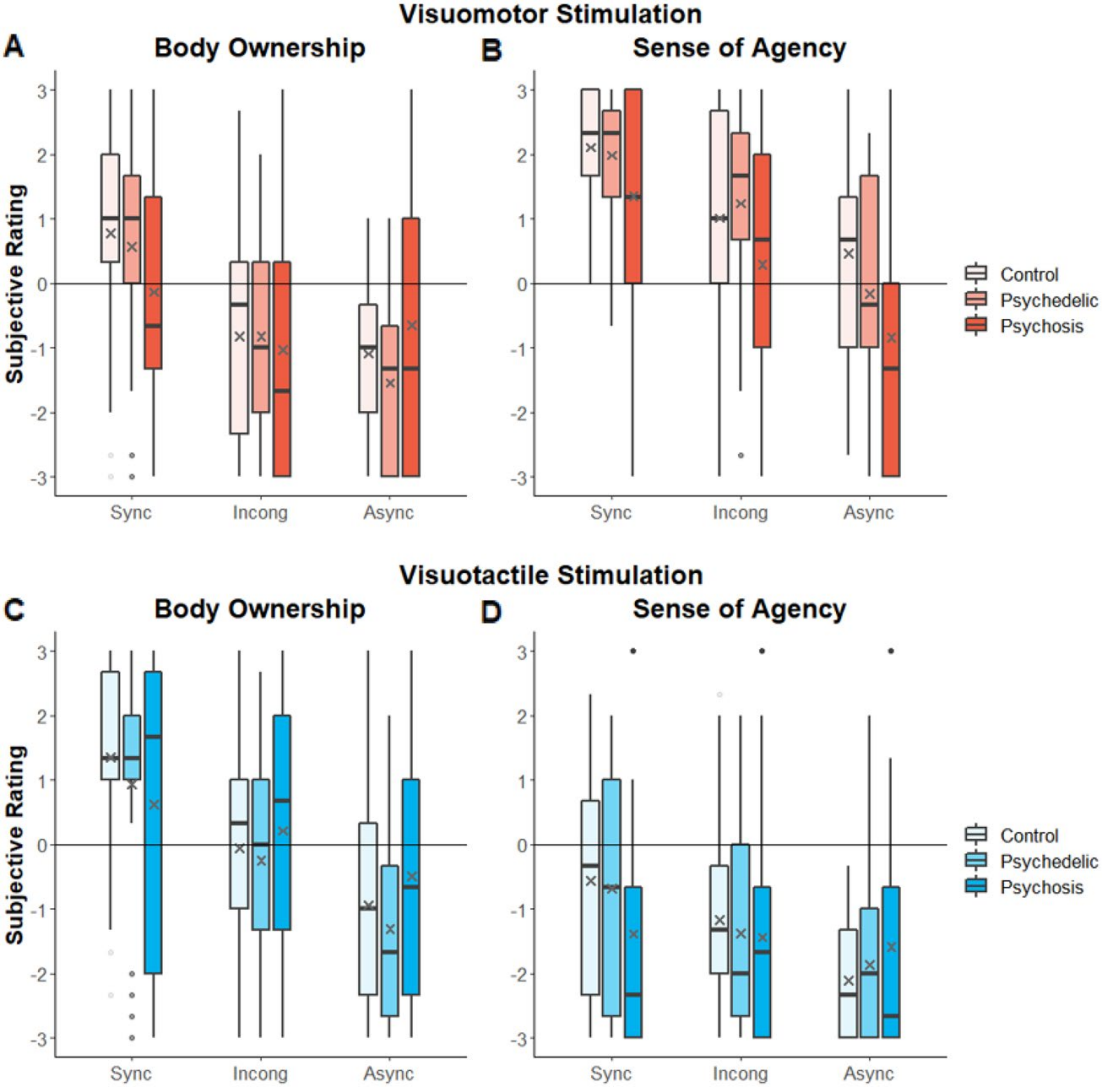
Behavioral Ratings. (**A**) Rating of Body Ownership in Visuomotor stimulation. (**B**) Ratings of Sense of Agency in Visuomotor stimulation (**C**) Ratings of Body Ownership in Visuotactile stimulation. (**D**) Ratings of Sense of Agency in Visuotactile stimulation. Gray ‘X’ at the boxplots represents the group’s mean.

As expected, Visuomotor stimulation induced an experience of Sense of Agency in the *Sync* condition (M = 1.81, SEM = 0.15, V = 2527, *p* < 0.001), the *Incong* condition (M = 0.85, SEM = 0.21, V = 2042, *p* < 0.001), where temporal coupling occurs, and was not significant in the *Async* condition (M = −0.18, SEM = 0.21, *p* > 0.05; see Fig. 3B). A main effect of Condition was found, (χ2 (2) = 52.4, *p* < 0.0001, Kendall’s W = 0.35), and post-hoc comparisons showed significant differences between all the combinations of conditions (*p* < 0.0001 for *Sync* vs. *Async* and *p* < 0.01 for the rest). Importantly, a main effect of Group was found (χ2 (2) = 6.68, *p* < 0.05, η^2^ = 0.02), and a post-hoc test revealed that this was driven by a significant difference between the *Psychosis* group and the *Control* group (*p* < 0.05) but was not significantly different between the *Psychosis* and *Psychedelic* groups (*p* = 0.057) and between the *Psychedelic* and *Control* groups (*p* = 0.567). A Bayesian analysis revealed moderate evidence supporting the lack of an effect between the *Control* and the *Psychedelic* groups (BF_01_ = 3.09). Despite lower Sense of Agency ratings of the *Psychosis* group, they were similarly affected by the different conditions in the Visuomotor stimulation, as can be seen by the lack of significant interaction between the Group and Condition factors (χ2 (2) = 1.54, *p* = 0.46). Notably, all the control statements ratings were negatively rated (*p* < 0.0001; Bonferroni Corrected). Thus, the *Psychosis* group showed reduced Sense of Agency compared to the *Control* group in the Visuomotor stimulation yet were comparably impacted by the modulation of anatomical congruence and temporal synchrony (Fig. 3B).

### Reduced body ownership during visuomotor stimulation in psychosis

As expected, Visuomotor stimulation induced an experience of Body Ownership in the *Sync* condition (M = 0.4, SEM = 0.21, V = 1665, *p* < 0.05), and a lower and non-significant in the *Incong* (M = −0.89, SEM = 0.19, V = 606, *p* = 1), and *Async* (M = −1.09, SEM = 0.2, V = 452, *p* = 1) conditions (Fig. 3A). A main effect of Condition was found (χ2 (2) = 39.3, *p* < 0.001, Kendall’s W = 0.26), and post-hoc comparisons showed significant differences between *Sync* vs. *Async* and *Sync* vs. *Incong* (*p* < 0.0001) and were not significant for the *Incong* vs. *Async* (*p* = 1). The main effect of Group was not significant (χ2 (2) = 1.34, p = 0.5). Notably, conditions affected the groups differently, as evidenced by a significant interaction between Condition and Group factors (χ2 (2) = 10.8, *p* < 0.001, η^2^ = 0.12). This interaction was driven by the lack of Body Ownership experience for the *Psychosis* group in the *Sync* condition (i.e., group’s mean is below 0). Importantly, the illusion strength of the *Healthy* group (*Psychedelic* and *Control*) was higher than the *Psychosis* group (*Healthy*: M = 2, SEM = 0.25; *Psychosis*: M = 0.52, SEM = 0.33; W = 916, *p* < 0.001). Additionally, a pairwise comparison between the typical difference of *Sync* and *Async* conditions was only significant in the *Control* (W = 252; *p* < 0.001) and *Psychedelic* (W = 290; *p* < 0.001) groups but was not significant in the *Psychosis* group (W = 107; *p* = 0.07). Nonetheless, a Bayesian analysis revealed moderate evidence supporting the lack of a group difference (main effect) between the *Control* and the *Psychedelic* groups (BF_01_ = 3.34) and between the *Control* and *Psychosis* groups (BF_01_ = 3.12). Notably, all the control statements ratings were negatively rated (*p* < 0.0001; Bonferroni Corrected). This lack of difference in the *Sync* and *Async* conditions, and diminished experience of Body Ownership illusion compared to the *Healthy* group, further supports our finding that the *Psychosis* group have impaired experience of Body Ownership under Visuomotor stimulation.

### Visuotactile stimulation induced similar body ownership across groups

The Visuotactile stimulation is similar to the classic Rubber Hand illusion, which is characterized by inducing Body Ownership over the rubber hand. As expected, the participants experienced Body Ownership over the rubber hand in the *Sync* condition (M = 0.97, SEM = 0.22, V = 2107.5, *p* < 0.001). The Body Ownership ratings were non-significant and gradually declined in the *Incong* (M = −0.03, SEM = 0.22, V = 1282, *p* = 0.49), and *Async* (M = −0.91, SEM = 0.19, V = 579, *p* = 1) conditions (Fig. 3C). This was supported by a significant main effect of Condition (χ2 (2) = 44.2, *p* < 0.0001, Kendall’s W = 0.29), and post-hoc comparisons which showed significant differences between all possible combinations of conditions (*p* < 0.0001 for *Sync* vs. *Async* and *p* < 0.01 for the rest). Comparing Body Ownership between groups, we did not find significant differences between *Control, Psychedelic, and Psychosis* groups across the different conditions. The main effect of Group was not significant (χ2 (2) = 1.35, *p* = 0.5). Finally, the interaction of Group and Condition was not significant (χ2 (2) = 5.58, *p* = 0.062, η2 = 0.05). A Bayesian analysis revealed anecdotal evidence supporting the lack of a difference between the *Control* and the *Psychedelic* groups (BF_01_ = 2.37) and moderate evidence between the *Control* and *Psychosis* groups (BF_01_ = 3.36). Notably, all the control statements ratings were negatively rated (*p* < 0.0001; Bonferroni Corrected). Thus, no significant differences between the *Psychosis*, *Psychedelic* and *Control* groups were found in the Visuotactile induction of illusory Body Ownership (Fig. 3C) and moderate evidence against such differences was present.

### Visuotactile stimulation did not induce sense of agency across groups

In general, the Visuotactile stimulation did not induce Sense of Agency experience over the rubber hand (*Sync:* M = −0.87, SEM = 0.21, V = 743, *p* = 1; *Incong:* M = −1.33, SEM = 0.18, V = 345, *p* = 1; *Async:* M = −1.85, SEM = 0.16, V = 154, *p* = 1; see Fig. 3D). A main effect of Condition was found (χ2 (2) = 21, *p* < 0.0001, Kendall’s W = 0.14). A post-hoc comparisons showed significant differences between the *Sync* vs. *Async* (*p* < 0.05) and nonsignificant for the rest (*p* > 0.05). The main effect of the Group was not significant (F(χ2) = 2.49, *p* = 0.28). Moreover, a Bayesian analysis revealed moderate evidence supporting the lack of a difference between the *Control* and the *Psychedelic* groups (BF_01_ = 3.12) and between the *Control* and *Psychosis* groups (BF_01_ = 2.56). A significant interaction between Group and Condition was found (χ2 (2) = 6.95, *p* < 0.05, η^2^ = 0.06). However, as mean ratings were below zero, we do not interpret this as inducing a Sense of Agency. All the control statements ratings were negatively rated (*p* < 0.0001; Bonferroni Corrected). Thus, in the absence of active movement, no experience of Sense of Agency over the rubber hand was formed, while the effect of condition was differently modulated between groups.

### Positive correlation between body ownership and sense of agency

**Figure 4:**
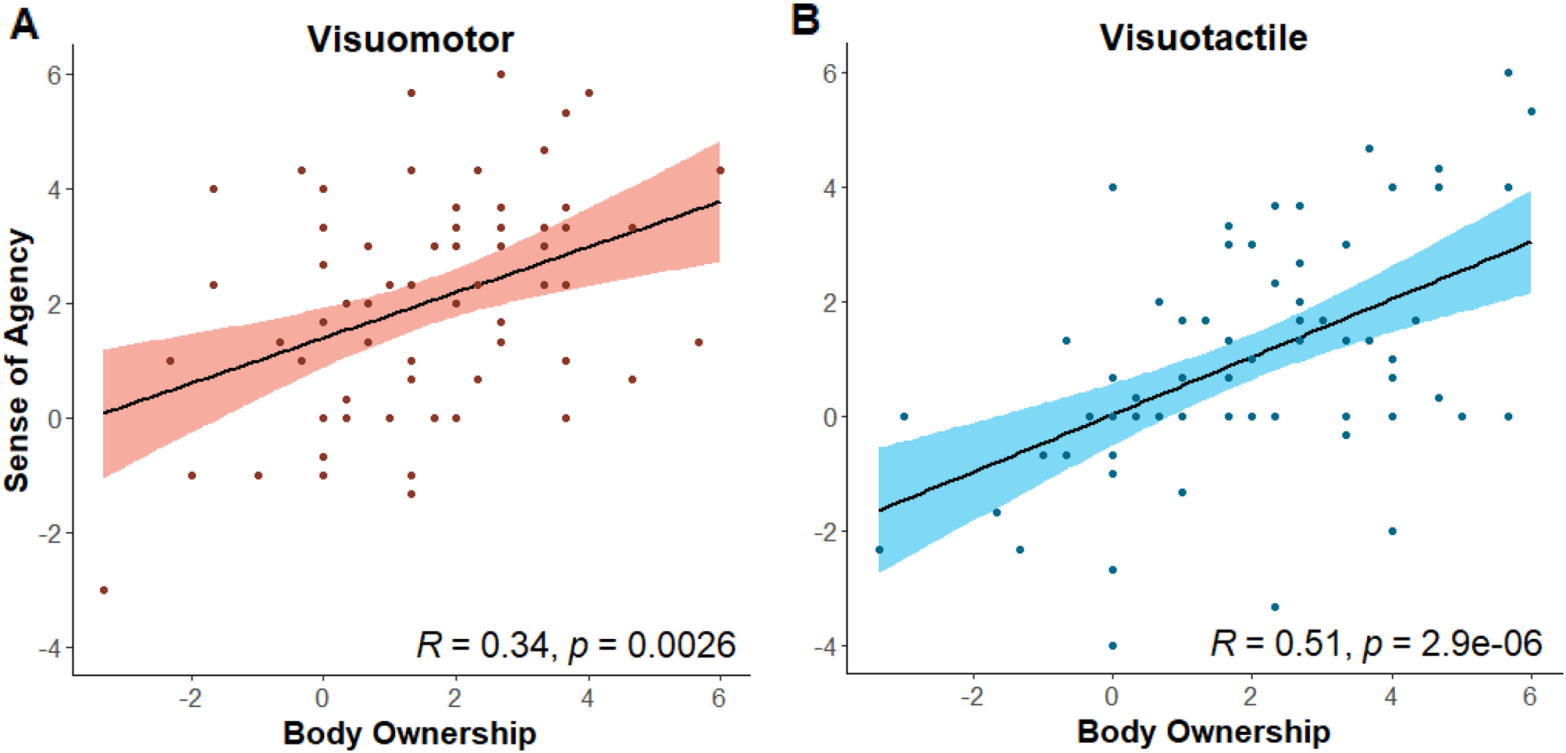
Behavioral Correlations of Body Ownership and Sense of Agency. Scatter plot with linear regression line for correlation between Sense of Agency and Body Ownership in Visuomotor stimulation (**A**) and Visuomotor stimulation (**B**).

We wanted to determine whether Body Ownership experience was correlated with Sense of Agency experience in both simulations (Visuomotor and Visuotactile). To examine it we calculated the subtraction between *Sync* and *Async* for Body Ownership and Sense of Agency for each stimulation. The differences from the subtraction represent the Body Ownership and Sense of Agency illusion strength. Body Ownership and Sense of Agency illusions in Visuomotor were significantly correlated (r = 0.34, *p* < 0.01; see Fig. 4A). Likewise, Body Ownership and Sense of Agency illusions in Visuotactile were also significantly correlated (*r* = 0.51, *p* < 0.001; see Fig. 4B). These results stand with the idea that when one experiences Sense of Agency over the rubber hand, one also tends to experience Body Ownership over the rubber hand.

## Discussion

The current study aimed at experimentally comparing how the bodily self is impacted in populations with modulations of the sense of self driven by psychotic or psychedelic experiences. Using the Moving Rubber hand Illusion, we tested both Body Ownership and the Sense of Agency in these populations. Our study revealed several interesting findings. First, as expected all populations showed modulations of Body Ownership using the classical Visuotactile stimulation. Second, the *Psychosis group* showed a reduced Sense of Agency during Visuomotor stimulation. Third, in the *Control* and *Psychedelic* groups, synchronous Visuomotor stimulation induced Body Ownership, which was absent in the *Psychosis* group. Finally, while the *Psychedelic* group reported altered self-experiences, we found no evidence for lasting modulations of the bodily self in this group.

As expected, synchronous Visuotactile stimulation induced illusory BO over the rubber hand. No significant differences were found between the *Control*, *Psychedelic,* and *Psychosis* groups and Bayesian analysis revealed moderate (i.e., *Control* vs. *Psychosis*) and anecdotal (i.e., *Control* vs. *Psychedelic*) evidence against such differences. This is in line with previous experimental and meta-analytical data showing no differences between *Control* and *Psychosis* groups in multisensory Body Ownership processing^97^. Similarly, this suggests that psychedelic experiences which often include striking modification of the phenomenological experiences of Body Ownership^29,100,121^, do not cause long-lasting alterations of multisensory bodily processing.

Previous theoretical and experimental work has highlighted abnormal Sense of Agency and predictive sensorimotor mechanisms as a central impairment across the schizophrenia spectrum^22,48,122–125^. For example, irregularities in Sense of Agency attribution have been shown in neurotypical individuals with high psychotypical scores^126,127^ and even in healthy individuals with a genetic propensity for schizophrenia^38^. In acute psychosis abnormalities in Sense of Agency processing are pronounced with both low accuracy in judgments and aberrant meta-cognition e.g.,^33,34,94^. Indeed, in a recent study embodied agency judgments allowed to classify psychotic participants with a high degree of accuracy^34^. The current results show reduced feeling of agency in the *Psychosis* group across all conditions indicating that they were affected by the conditions but had diminished sensation of control over the movements. This finding is in line with the passivity experiences in schizophrenia, which involve a reduction in Sense of Agency^128–130^. Furthermore, previous studies using body illusions, revealed that individuals on the schizophrenia spectrum experience a reduction in Sense of Agency similar to healthy individuals when exposed to Visuotactile or Visuomotor asynchronous stimulations, as evidenced by decreased Sense of Agency compared to synchronous stimulations^128,131,132^.

Synchronous Visuomotor stimulation also induced sensations of Body Ownership in the *Control* and *Psychedelic* groups but not the *Psychosis* group. Several studies have shown that Sense of Agency and Body Ownership are often correlated^89,92,93^, suggesting that they impact each other. Reduced Body Ownership during synchronous Visuomotor stimulation suggests that aberrant sensations of bodily self in psychosis may stem not from deficits in Body Ownership processing per se ^97^ but rather from reduced Sense of Agency which in turn impacts the feeling of ownership over body parts. Thus, while the *Psychosis* group showed typical Body Ownership and Sense of Agency in the Visuotactile conditions, these experiences were reduced specifically in Visuomotor conditions. This result joins previous evidence that the experience of the bodily self is disrupted in psychosis patients. Nonetheless, our findings suggest that these altered bodily experiences in psychosis patients are not a global impairment, but rather related to aberrant processing of volitional actions^33,34,48,128,133^.

Importantly, our findings highlight that while psychedelic experiences are associated with dramatic changes in the sense of self, including the bodily self^29,100^, these were not associated with experimentally induced changes in Body Ownership or Sense of Agency. In fact, across all experimental conditions the *Psychedelic* group’s results were nearly identical to those of the *Control* group. This finding is especially salient considering the clear differences in subjective experiences of modifications of the self between these groups (See Fig. 2 and Supplementary Table S2). This suggests that while some changes to the sense of self induced by psychedelic experiences are retained at explicit levels of the self-model, fundamental sensorimotor processing of the bodily self is not altered in an enduring manner. Thus, our data provide evidence against psychotomimetic approaches to psychedelic experiences at least for bodily self-processing, in such that the *Psychosis* and *Psychedelic* groups showed no similarities in their results.

While considering that this is a novel study which must be replicated, we note several interesting implications arising from our findings. First, no evidence for modulations of the bodily self were shown in the psychedelic group. This suggests that long lasting changes in the sense of self are not evident at the level of the bodily self. Furthermore, psychosis patients showed atypical bodily self only in conditions including volitional action. This implicates Sense of Agency and action related processing as the primary mechanisms driving abnormal sense of bodily self in psychosis^34,97,134^. These findings may guide future studies in psychedelics to focus on higher levels of self-related processing e.g.,^9,135^, while targeting volitional action as a critical subject in the study of psychosis ^136–138^.

## Limitations

Our study has several limitations which should be addressed. Due to the age limits of the population at the mental health center, the psychotic group was on average older than the other groups. Nonetheless, age differences were previously not found to affect the illusion strength^139^. Since the experiment was conducted in a male only ward of the Beer Yaacov-Ness Ziona mental health center, the *Psychosis* group consisted only of men. While Psychosis participants were screened for current or previous substance addictions, and none had a history of substance induced psychosis, we didn’t specifically inquire about psychedelic use. It is thus possible that some of these patients had previously experienced psychedelics. Furthermore, due to time constraints as well as the limited reliability of self-report questionnaires in psychosis we opted to assess the sense of self using the PANSS in this group. This prevented direct comparisons of questionnaire scores with the other groups. We didn’t include a passive movement condition in this study as we had a limited duration for our experiment with the psychosis group. This condition could have allowed additional insight into the effects of volitional action on the sense of self e.g.,^92,93^. Additionally, we didn’t collect proprioceptive drift measures, which have often been used as an implicit measure of the Rubber Hand illusion e.g.,^92,93^. As recent studies have found dissociations between proprioceptive drift and body ownership measurements^140–144^ and given the time limitations with the clinical cohort we decided not to include this measure in the design. Finally, due to ethical and experimental limitations, we could not collect psychedelic participants in the acute stage of the psychedelic experience, limiting any inferences regarding their acute effects. Further studies are needed to explore how different psychedelic compounds impact the sense of self, during and after the psychedelic experience.

## Conclusions

Our study shows that psychosis impacts the bodily self with diminished agency and body ownership during volitional actions. Contrarily, while substantial psychedelic use was associated with reported enduring changes in the experience of self, it showed no impact on processing of the bodily self. Our results suggest that even considerable psychedelic use doesn’t alter multisensory bodily processing underlying the sense of self, but may manifest changes in more explicit, narrative models of the self.

### Supplementary Information

Supplementary Information.

## Data Availability

The datasets used and/or analyzed during the current study is available from the corresponding author on reasonable request.
