# Supplementary material for: The bodily self from psychosis to psychedelics
Source: Sci Rep. 2023 Dec 1;13:21209. doi: 10.1038/s41598-023-47600-z (PMC10692325; doi:10.1038/s41598-023-47600-z)
Supplement: Supplementary file 1 — Supplementary Information. [file 41598_2023_47600_MOESM1_ESM.docx]

**Supplementary Materials**

| **Body Ownership** | I felt as if I was looking at my own hand |
| --- | --- |
|  | I felt as if the rubber hand was part of my body |
|  | I felt as if the rubber hand was my hand |
| **Body Ownership Control** | It seems as if I had more than one right hand |
|  | It felt as if I no longer had a right hand as if my right hand had disappeared |
|  | I felt as if my real hand was turning rubbery |
| **Sense of Agency** | I felt as if I could cause movements if the rubber hand |
|  | I felt as if I could control movements if the rubber hand |
|  | The rubber hand was obeying my will and I can make it mine just like I want it |
| **Sense of Agency Control** | I felt as if the rubber hand was controlling my will |
|  | It seemed as if the rubber hand had a will of its own |
|  | I felt as if the rubber hand was controlling me |

**Table S1 - MRHI Questionnaire.** Statements used in the experiment to measure the experience of ownership and agency with their respective control categories.

**Assessment of participant characteristics**

**Subsequent changes in the psychedelic group's sense of self**

To assess subsequent changes in the *Psychedelic* group's sense of self, we constructed a questionnaire regarding altered self-experiences consisting of 18 statements. These statements were gathered from several well-established questionnaires that examine the frequency and duration of experiences such as: depersonalization symptoms over the last six months, ego-dissolution experiences occasioned by psychedelics substance, measurements of internal and external unity, and transcendence of time and space. Participants were asked to rate the degree to which each statement reflected their own experiences using a scale of 1 ("Do not agree") to 10 ("Agree"). The statements were driven from several questionnaires: Ego-Dissolution Inventory (EDI), Cambridge Depersonalization Scale (CDS), and the Pahnke-Richards Mystical Experience Questionnaire. To estimate if the *Psychedelic* group had greater ratings than *Control* group, we performed a Mann–Whitney test for each statement.

| **Questionnaire** | **Statements** | **Statics T(48)** |
| --- | --- | --- |
| **Cambridge Depersonalisation Scale (CDS)** | Whilst doing something I have the feeling of being a ‘detached observer’ of myself | Control: Mean = 3.88, SD = 2.53 Psychedelic: Mean = 6.92, SD = 2.73 W = 495 p < 0.001 |
|  | Parts of my body feel as if they didn’t belong to me | Control: Mean = 2.56, SD = 2.29 Psychedelic: Mean = 4.84, SD = 3.65 W = 410.5 p < 0.05 |
|  | I have the feeling of not having any thoughts at all, so that when I speak it feels as if my words were being uttered by an ‘automaton’ | Control: Mean = 3.36, SD = 2.59 Psychedelic: Mean = 5.44, SD = 2.95 W = 435.5 p < 0.01 |
|  | It seems as if things that I have recently done had taken place a long time ago. For example, anything which I have done this morning feels as if it were done weeks ago | Control: Mean = 5.8, SD = 3.13 Psychedelic: Mean = 6.36, SD = 3.3 W = 351.5 p = 0.22 |
|  | I cannot feel properly the objects that I touch with my hands for it feels as if it were not me who was touching it | Control: Mean = 2.04, SD = 2.13 Psychedelic: Mean = 4, SD = 2.88 W = 472 p < 0.001 |
|  | While fully awake I have ‘visions’ in which I can see myself outside as if I were looking at my image in a mirror | Control: Mean = 2.76, SD = 2.65 Psychedelic: Mean = 5.36, SD = 3.05 W = 486.5 p < 0.001 |
|  | When I move it doesn’t feel as if I were in charge of the movements so that I feel ‘automatic’ and mechanical as if I were a ‘robot’ | Control: Mean = 3.52, SD = 2.53 Psychedelic: Mean = 4.24, SD = 2.74 W = 365.5 p = 0.15 |
|  | I have to touch myself to make sure that I have a body or a real existence | Control: Mean = 2.2, SD = 2.39 Psychedelic: Mean = 4.72, SD = 3.44 W = 452 p < 0.01 |
|  | Familiar voices (including my own) sound remote and unreal | Control: Mean = 2.24, SD = 2.5 Psychedelic: Mean = 6.12, SD = 2.62 W = 541.5 p < 0.0001 |
|  | I feel detached from memories of things that have happened to me – as if I had not been involved in them | Control: Mean = 3.2, SD = 2.88 Psychedelic: Mean = 5.32, SD = 3.33 W = 427.5 p < 0.05 |
| **Ego-Dissolution Inventory**  **(EDI)** | I experienced a disintegration of my ‘’self’’ or ego | Control: Mean = 3.52, SD = 2.22 Psychedelic: Mean = 7.76, SD = 2.67 W = 549 p < 0.0001 |
|  | I felt at one with the universe | Control: Mean = 3.52, SD = 2.96 Psychedelic: Mean = 8.6, SD = 2.2 W = 550 p < 0.0001 |
|  | I felt more important or special than others | Control: Mean = 5.36, SD = 2.75 Psychedelic: Mean = 6.24, SD = 2.86 W = 373 p = 0.12 |
|  | All notion of self and identity dissolved away | Control: Mean = 3, SD = 2.6 Psychedelic: Mean = 6.52, SD = 3.11 W = 515 p < 0.0001 |
| **Pahnke-Richards Mystical Experience Questionnaire (MEQ)** | Freedom from the limitations of your personal self and feeling a unity or bond with what was felt to be greater than your personal self | Control: Mean = 4.04, SD = 3.38 Psychedelic: Mean = 8.96, SD = 1.76 W = 537.5 p < 0.0001 |
|  | Sense of being “outside of” time, beyond past and future | Control: Mean = 2.64, SD = 3.13 Psychedelic: Mean = 8.48, SD = 2.4 W = 562 p < 0.0001 |
|  | Loss of usual awareness of where you were | Control: Mean = 3.28, SD = 2.77 Psychedelic: Mean = 7.88, SD = 1.92 W = 555.5 p < 0.0001 |
|  | Experience of the fusion of your personal self into a larger whole | Control: Mean = 2.12, SD = 2.12 Psychedelic: Mean = 7.76, SD = 2.8 W = 581 p < 0.0001 |

**Table S2 - Altered self-experiences** **questionnaire.** The statements and their differences in self-related experiences.

| **Stimulation** | **Experience** | **Comparison** | **Cauchy 0.353** | **Cauchy 0.707** | **Cauchy 1.414** |
| --- | --- | --- | --- | --- | --- |
| Visuomotor | Sense of Agency | *Control* vs. *Psychedelic* | 1.97 | 3.09 | 4.44 |
| Visuomotor | Body Ownership | *Control* vs. *Psychedelic* | 1.94 | 3.34 | 4.69 |
|  |  | *Control* vs. *Psychosis* | 1.94 | 3.12 | 5.75 |
| Visuotactile | Body Ownership | *Control* vs. *Psychedelic* | 1.53 | 2.37 | 3.18 |
|  |  | *Control* vs. *Psychosis* | 2.00 | 3.36 | 6.43 |
| Visuotactile | Sense of Agency | *Control* vs. *Psychedelic* | 1.81 | 3.12 | 4.30 |
|  |  | *Control* vs. *Psychosis* | 1.65 | 2.56 | 4.57 |

**Table S3 - Robustness of Base factors for null results.** Reports on the robustness of Base Factor findings are calculated using a default prior (Cauchy = 0.707), a wide prior (Cauchy = 1.414), and a narrow prior (Cauchy = 0.3535) and presented as Base factors of exclusion (BF_01_).

**
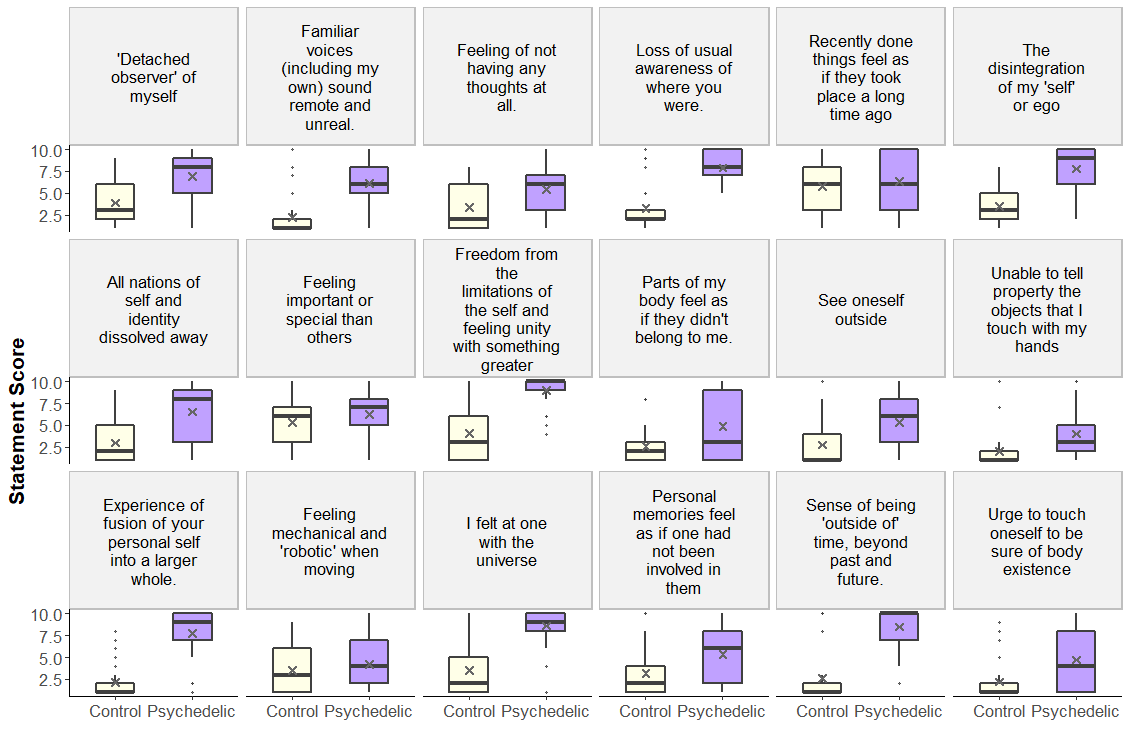
**

**Figure S1 - Detailed ratings in self-experience questionnaire.** Statements scores are presented for each question. Gray ‘X’ at the boxplots represents the group’s mean.

| **Diagnosis** | **N** | **Mean PANSS(±SD)** |
| --- | --- | --- |
| Schizophrenia | 17 | 75.64(±15.74) |
| Schizoaffective disorder | 7 | 66(±7.6) |
| Severe depressive episode with psychotic symptoms | 1 | 62 |

**Table S4 - Clinical characteristics of psychosis patients.** PANSS = Positive and Negative Syndrome Scale

**
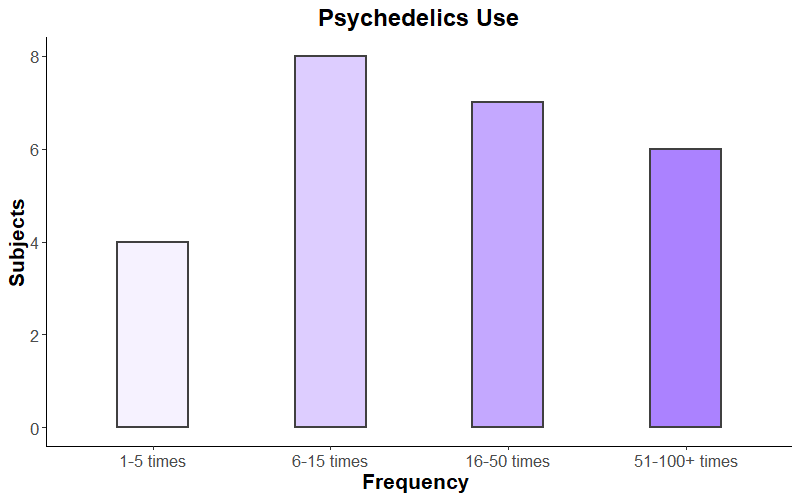
**

**Figure S2 - Frequency usage of psychedelic substances**. The Y-axis is the number of subjects according to the number of experiences with psychedelics throughout life (X-axis) in the *Psychedelic* group.

**
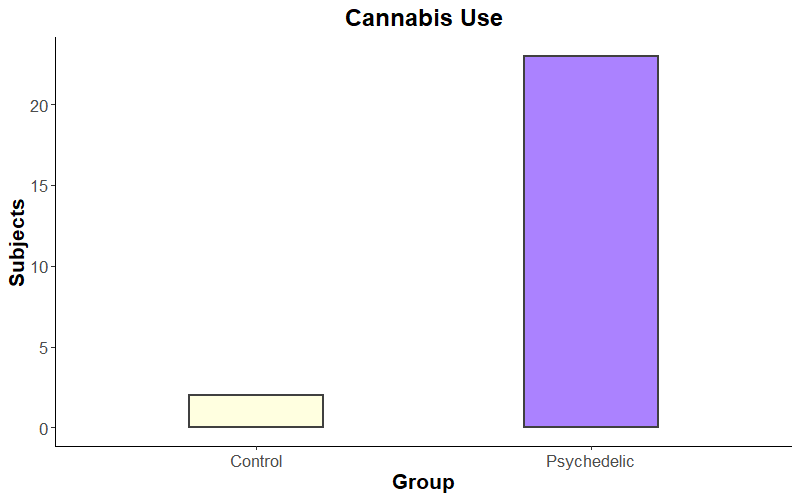
**

**Figure S3 - Cannabis use in *Control* and *Psychedelic* groups.** The Y-axis is the number of subjects that declared use of Cannabis in the *Control* versus *Psychedelic* groups.


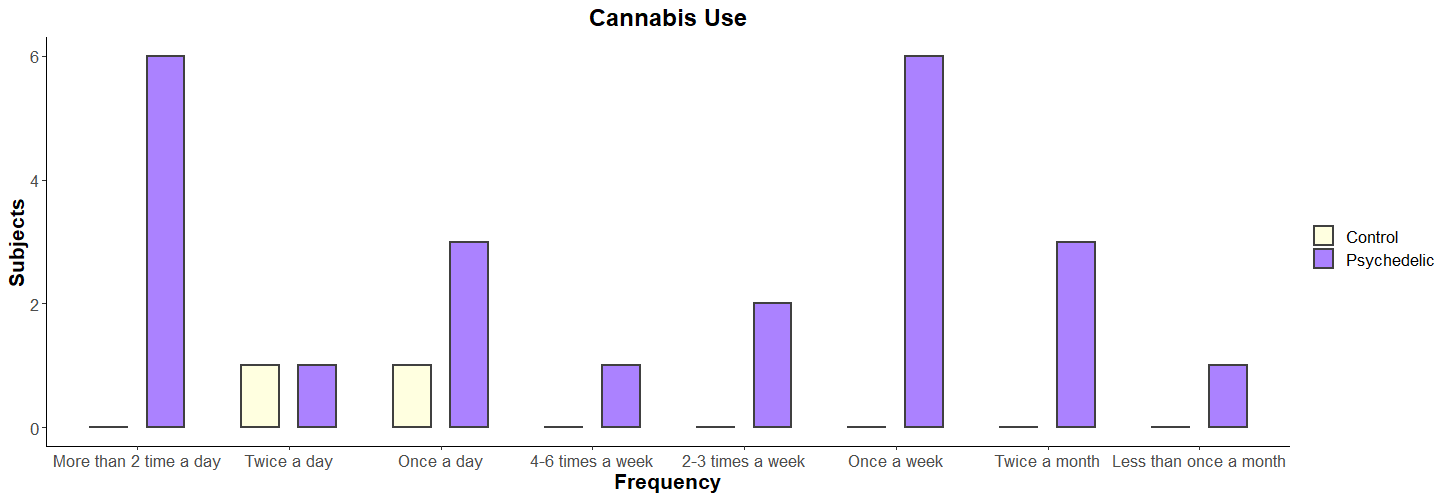


**Figure S4 - Frequency usage of Cannabis in *Control* and *Psychedelic* groups.** The Y-axis is the number of subjects according to the frequency of their Cannabis use (X-axis) in the *Control* versus *Psychedelic* groups.

**
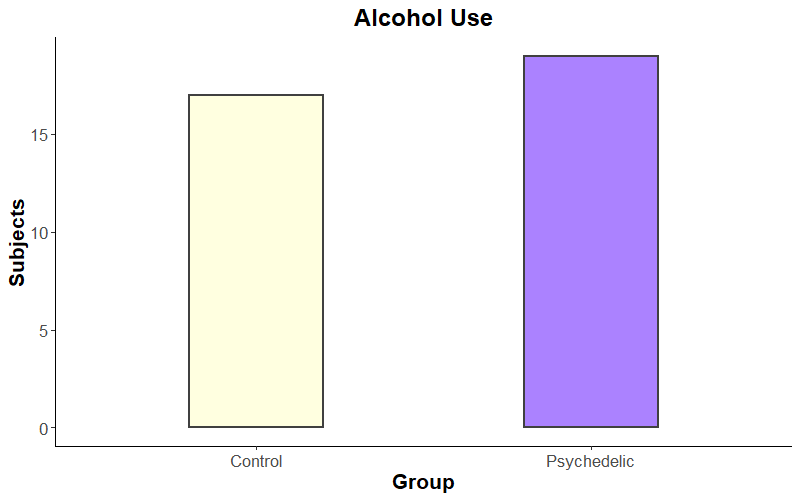
**

**Figure S5 - Alcohol use in *Control* and *Psychedelic* groups.** The Y-axis is the number of subjects that declared use of alcohol in the *Control* versus *Psychedelic* groups.


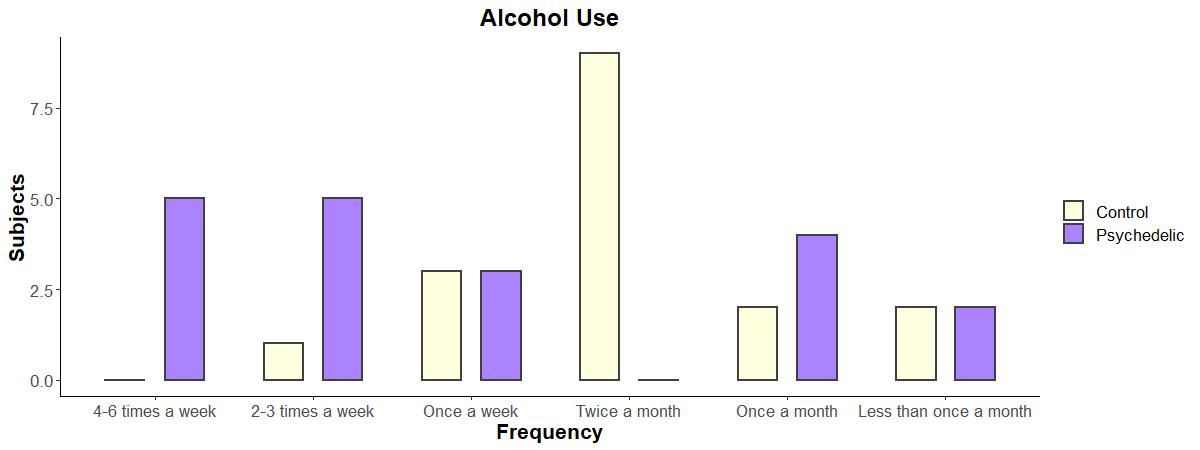


**Figure S6 - Frequency usage of Cannabis in *Control* and *Psychedelic* groups.** The Y-axis is the number of subjects according to the frequency of their alcohol use (X-axis) in the *Control* versus *Psychedelic* groups.
